# Insulin stimulation of Akt/PKB phosphorylation in the placenta of preeclampsia patients

**DOI:** 10.1590/S1516-31802011000600004

**Published:** 2011-12-01

**Authors:** Gustavo Dias Ferreira, Rafael Bueno Orcy, Sérgio Hofmeister Martins-Costa, José Geraldo Lopes Ramos, Ilma Simoni Brum, Helena von Eye Corleta, Edison Capp

**Affiliations:** I MSc, Molecular, Endocrine and Tumor Biology Laboratory, Universidade Federal do Rio Grande do Sul (UFRGS), and PhD Student in Gynecology and Molecular Obstetrics Laboratory, Research Center, Hospital de Clínicas de Porto Alegre, Porto Alegre, Rio Grande do Sul, Brazil.; II PhD. Physiologist, Molecular, Endocrine and Tumor Biology Laboratory, Universidade Federal do Rio Grande do Sul (UFRGS), and Researcher in Gynecology and Molecular Obstetrics Laboratory, Research Center, Hospital de Clínicas de Porto Alegre, Porto Alegre, Rio Grande do Sul, Brazil.; III MD, PhD. Adjunct Professor, Gynecology and Obstetrics Service, Hospital de Clínicas de Porto Alegre, and Department of Gynecology and Obstetrics, School of Medicine, Universidade Federal do Rio Grande do Sul (UFRGS), Porto Alegre, Rio Grande do Sul, Brazil.; IV MD, PhD. Associate Professor, Gynecology and Obstetrics Service, Hospital de Clínicas de Porto Alegre, and Department of Gynecology and Obstetrics, School of Medicine, Universidade Federal do Rio Grande do Sul (UFRGS), Porto Alegre, Rio Grande do Sul, Brazil.; V MD, PhD. Associate Professor, Department of Physiology, Molecular, Endocrine and Tumor Biology Laboratory, Universidade Federal do Rio Grande do Sul (UFRGS), Gynecology and Molecular Obstetrics Laboratory, Research Center, Hospital de Clínicas de Porto Alegre, Porto Alegre, Rio Grande do Sul, Brazil.; VI MD. Associate Professor, Gynecology and Obstetrics Service, Hospital de Clínicas de Porto Alegre, and Department of Gynecology and Obstetrics, School of Medicine, Universidade Federal do Rio Grande do Sul (UFRGS), Porto Alegre, Rio Grande do Sul, Brazil.; VII MD, PhD. Associate Professor, Department of Gynecology and Obstetrics, Universidade Federal do Rio Grande do Sul (UFRGS), and Coordinator of Master's degree program in Biological Sciences (Physiology), Molecular, Endocrine and Tumor Biology Laboratory, UFRGS, Porto Alegre, Rio Grande do Sul, Brazil.

**Keywords:** Pre-eclampsia, Placenta, Insulin resistance, Receptor, insulin, Proto-oncogene proteins c-akt, Pré-eclâmpsia, Placenta, Resistência à insulina, Receptor de insulina, Proteínas proto-oncogênicas c-akt

## Abstract

**CONTEXT AND OBJECTIVE:**

Preeclampsia is a multi-systemic disease and one of the most frequent severe health problems during pregnancy. Binding of insulin triggers phosphorylation and activates cytoplasmic substrates such as phosphatidylinositol 3 kinase (PI3K). Phosphorylation of membrane phosphoinositide 2 (PIP2) to phosphoinositide 3 (PIP3) by PI3K starts Akt/PKB activation. Defects in phosphorylation of the insulin receptor and its substrates have an important role in insulin resistance. Studies have shown that insulin resistance is associated with preeclampsia and its pathophysiology. The aim here was to investigate insulin stimulation of the Akt/PKB pathway in the placenta, in normal and preeclampsia parturients.

**DESIGN AND SETTING:**

Cross-sectional study in a tertiary public university hospital.

**METHODS:**

Placentas were collected from 12 normal and 12 preeclampsia patients. These were stimulated and analyzed using Western blot to quantify the Akt/PKB phosphorylation.

**RESULTS:**

The insulin stimulation was confirmed through comparing the stimulated group (1.14 ± 0.10) with the non-stimulated group (0.91 ± 0.08; P < 0.001). The phosphorylation of Akt/PKB did not differ between the placenta of the normal patients (1.26 ± 0.16) and those of the preeclampsia patients (1.01 ± 0.11; P = 0.237).

**CONCLUSIONS:**

*In vitro* insulin stimulation of the human placenta has been well established. There was no difference in Akt/PKB phosphorylation, after stimulation with insulin, between placentas of normal and preeclampsia patients. Nevertheless, it cannot be ruled out that the Akt/PKB signaling pathway may have a role in the pathophysiology of preeclampsia, since the substrates of Akt/PKB still need to be investigated.

## RESUMO

**CONTEXTO E OBJETIVO:**

Pré-eclâmpsia (PE) é uma doença multissistêmica das mais frequentes e graves durante a gestação. A ligação da insulina inicia a fosforilação e ativação de substratos citoplasmáticos, tais como fosfatidil-inositol 3 quinase (PI3K). A fosforilação do fosfoinositol 2 (PIP2) da membrana em fosfoinosiltol 3 (PIP3) pela PI3K inicia a ativação da Akt/PKB. Defeitos na fosforilação do receptor de insulina e seus substratos têm papel importante na resistência à insulina. Estudos demonstraram que resistência à insulina está associada com pré-eclâmpsia e sua patofisiologia. O objetivo foi investigar a via de estimulação com insulina da Akt/PKB em placenta de parturientes normais e com pré-eclampsia.

**TIPO DE ESTUDO E LOCAL:**

Estudo do tipo transversal em um hospital universitário público de nível terciário.

**MÉTODOS:**

Vinte e quatro placentas (12 normais, 12 com PE) foram coletadas, estimuladas e analisadas por *Western blot* para quantificar a fosforilação da Akt/PKB.

**RESULTADOS:**

A estimulação com insulina foi confirmada comparando os grupos estimulados (1,14 ± 0,10) e não estimulados (0.91 ± 0.08; P < 0.001). A fosforilação de Akt/PKB não foi diferente na placenta de pacientes normais (1,26 ± 0,16) e com PE (1,01 ± 0,11; P = 0,237).

**CONCLUSÕES:**

A estimulação *in vitro* da placenta humana com insulina foi bem estabelecida. Não houve diferença na fosforilação da Akt/PKB após estimulação em placentas de pacientes normais e PE. Contudo, não é possível descartar a participação desta via de sinalização na patofisiologia da PE, uma vez que os substratos da Akt/PKB ainda precisam ser investigados.

## INTRODUCTION

Preeclampsia is a multi-systemic disease and is considered to be one of the most significant health problems in pregnancy. It occurs mainly in nulliparous women, particularly after the 20^th^ week of gestation, and most frequently near delivery.^1^ It is diagnosed when the patient presents gestational hypertension associated with proteinuria, vasoconstriction of the maternal vascular bed and, consequently, increased vascular resistance.^2-4^ The incidence ranges from 2-5% to more than 10% of pregnancies in developing countries, where prenatal care is still inadequate.^1^ It can affect both maternal and fetal health,^5^ leading to fetal growth restriction, prematurity and, in severe cases, maternal and perinatal death.^6-8^ Studies have shown that insulin resistance is associated with preeclampsia and contributes towards its pathophysiology.^9^

The insulin receptor belongs to the family of tyrosine kinases (RTKs). The binding of insulin triggers phosphorylation and activates cytoplasmic substrates such as phosphatidylinositol 3 kinase (PI3K). Phosphorylation of membrane phosphoinositide 2 (PIP2) to phosphoinositide 3 (PIP3) by PI3K starts Akt/PKB activation.^10,11^ Defects in phosphorylation of the insulin receptor and its substrates, and non-activation of PI3K-Akt/PKB has an important role in developing insulin resistance.^10,12^ If not activated, the Akt/PKB pathway will also not phosphorylate its substrates, which participate in various cell functions, such as control of metabolism, survival, glucose uptake, proliferation, growth and angiogenesis.^13^

## OBJECTIVE

The aims of this study were to achieve *in vitro* insulin stimulation of the human placenta and to investigate the expression of the protein Akt/PKB in the baseline state and after stimulation, in the placentas of normal and preeclampsia patients.

## METHODS

A cross-sectional study with control group was performed. Twenty-four women participated, including 12 patients who presented a medical diagnosis of preeclampsia and 12 normotensive pregnant women (control group). The samples were divided into four groups: stimulated controls, non-stimulated controls, stimulated preeclampsia patients and non-stimulated preeclampsia patients. Information such as age, use of hormonal medication, family history of diabetes mellitus, gynecological and obstetric history, fasting glucose, blood pressure, proteinuria, glucose tolerance test of the mother and gestational age was gathered before the birth. This study was submitted to and approved by the Research Ethics Committee of the Research and Postgraduate Program Group of Hospital de Clínicas de Porto Alegre (GPPG 08-124).

### Sample preparation

Approximately 30 g of placenta were obtained immediately after cesarean sections. The samples were washed with phosphate-buffered saline (PBS) (4 °C) to remove excess blood and were taken to the laboratory for preparation and stimulation with insulin.

The preparation of the placenta was performed in accordance with Klein et al.,^14^ with modifications. The tissue was weighed in the laboratory, separated from blood vessels and cut in slices. One gram of tissue was incubated in 5 ml of bovine serum albumin (BSA) buffer: 32 mM of HEPES, 195 mM of NaCl, 7.2 mM of KCl, 1.8 mM of KH_2_PO_4_, 8.3 mM of glucose and 1% albumin in distilled H_2_O. Briefly, the placental tissue was digested with collagenase type I (Gibco, Invitrogen Corporation) and incubated at 37 °C under orbital stirring (100 rpm) for 45 minutes. After this, a 250 mm filter was used to separate out and remove large particles and remaining fibrin. For cell separation, the sample was divided into two 50 ml tubes and centrifuged at 2000 x g for 10 minutes at 30 °C. After the supernatant had been discarded, a small portion of the sample was viewed under a microscope to observe the viability of the cells for stimulation. The cells were then homogenized with 0.5 ml of PBS. These samples were transferred to two 1.5 ml tubes, which were incubated with 0.5 ml of a stimulation buffer containing 50 mM of Tris-HCl (pH 7.4), 0.01% BSA, 1 mM of ATP, 2 mM of MgCl_2_, 1 mM of EDTA, 5 mM of sodium pyrophosphate, 1 mM of sodium orthovanadate and 50 mM of sodium fluoride, with and without insulin [10^-7^M] for eight minutes at 37 °C.^9^ For analysis, placental pieces were pooled and homogenized. Protein concentrations were measured using Bradford's method.^15^

The samples were analyzed using Western blot.^16,17^ Samples of 60 μg of protein (per lane) were loaded onto 10% acrylamide gel. The proteins were transferred to nitrocellulose membranes by means of a semidry system. Rabbit polyclonal antibodies (Santa Cruz, California, United States) were used: total anti-Akt (sc-8312) and phosphor-Akt (Ser473) (sc-7985).

### Statistical analysis

Statistical analysis was performed by means of the Statistical Package for the Social Sciences (SPSS) 15.0 software. The data were tested using Student's t test for paired samples and independent samples, for parametric variables, and were shown as means ± standard deviations (SD). The significance level was taken to be P < 0.05.

## RESULTS

The patients with preeclampsia fulfilled the diagnostic criteria, presenting proteinuria over 300 mg/dl and hypertension. In the normotensive group, there were no patients with proteinuria over 300 mg/dl. The normotensive and preeclampsia groups did not differ significantly in age (26.8 ± 4.1 versus 27.8 ± 8.2; P = 0.714), body mass index (BMI) (28.4 ± 3.5 versus 31.3 ± 4.1; P = 0.08) or number of pregnancies (2.5 ± 2.1 versus 1.8 ± 1.1; P = 0.303) (Table 1). The mean gestational age was approximately four weeks shorter in the preeclampsia group (35.6 ± 0.5 versus 39.1 ± 0.4; P < 0.001), while the fasting glucose levels (mg/dl) were significantly higher in the preeclampsia group (91.58 ± 2.83 versus 76.00 ± 2.39; P < 0.001). Diastolic and systolic blood pressures were significantly higher in the preeclampsia patients (P < 0.001) (Table 1).

**Table 1.** Clinical characteristics of women with preeclampsia and control subjects

	Normal	PE	P
Age (years)	26.8 ± 4.1	27.8 ± 8.2	0.714
BMI (kg/m²)	28.4 ± 3.5	31.3 ± 4.1	0.080
Gestational age (weeks)	39.1 ± 0.4	35.6 ± 0.5	< 0.001*
Systolic blood pressure (mmHg)	118.5 ± 10	154.8 ± 10	< 0.001*
Diastolic blood pressure (mmHg)	70.6 ± 6.4	97.5 ± 5.9	< 0.001*
Number of pregnancies	2.5 ± 2.1	1.8 ± 1.1	0.303
Fasting glucose (mg/dl)	76.0 ± 2.3	91.5 ± 2.8	< 0.001*

PE = preeclampsia; BMI = body mass index.

* Student's t test.

### Sample stimulation

**Figure 1.**
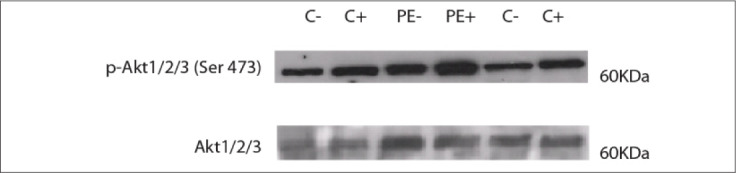
Representative Western blot analysis on placenta samples from patients with preeclampsia (PE) and normal controls (C): sample stimulated with insulin (+); sample not stimulated with insulin (-).

Sample stimulation was assessed by comparing the expression of phosphor-Akt (Ser473) in samples stimulated with insulin (+) and not stimulated (-), both in the control group and in the preeclampsia group. The values were normalized according to total anti-Akt protein expression (60 kDa) (Figure 1).

**Figure 2.**
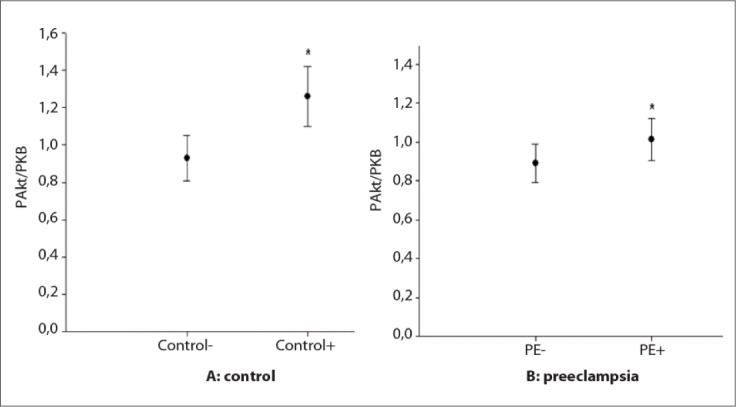
Expression of phosphor-Akt (Ser473) in stimulated and non-stimulated samples. In the control group (A), 0.93 ± 0.12 for the non-stimulated samples (-) and 1.26 ± 0.16 for the stimulated samples (+) (P < 0.001). In the preeclampsia group (B), 0.89 ± 0.11 for the non-stimulated samples (-) and 1.01 ± 0.11 for the stimulated samples (+) (P = 0.001).

In the control group (n = 12), the expression of phosphor-Akt (Ser473) was 1.26 ± 0.16 for samples stimulated with insulin (+) and 0.93 ± 0.12 for samples not stimulated (-), with a significant difference of P < 0.001. In the PE group (n = 12), the expression was 1.01 ± 0.01 for samples stimulated with insulin and 0.89 ± 0.11 for samples not stimulated, with a significant difference of P = 0.001, thus confirming that the samples had been stimulated (Figure 2).

### Protein expression in the preeclampsia group and control group

To compare the expression of Akt/PKB between the preeclampsia and control group, the analyses were performed in two states: baseline state (no stimulation with insulin) and stimulated state.

**Figure 3.**
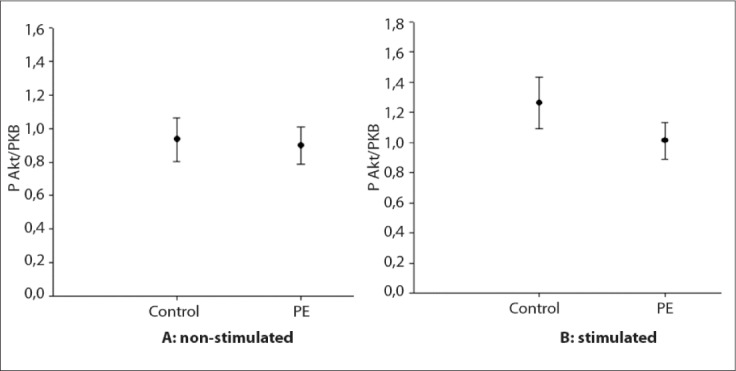
Expression of phosphorylation of Akt/PKB compared between the non-stimulated control group and preeclampsia group (A) (P = 0.828), and between the stimulated control group and preeclampsia group (B) (P = 0.237).

In the baseline state, the expression of phosphor-Akt (Ser473) was 0.93 ± 0.13 in the control group (n = 12) and 0.89 ± 0.11 in the preeclampsia group (n = 12) (P = 0.82) (Figure 3A). Among the samples stimulated with insulin, the expression in the control group (n = 12) was 1.26 ± 0.16 and in the preeclampsia group (n = 12), it was 1.01 ± 0.11 (P = 0.23) (Figure 3B). There was no statistically significant difference between the preeclampsia group and the control group, either in the baseline state or in the stimulated state.

## DISCUSSION

Preeclampsia occurs frequently during pregnancy, and it is a multi-system disease of unknown etiology.^3,18^ Insulin resistance and obesity have been defined as risk factors for the development of this disease.^9,19,20^

In the present study, age and BMI did not differ between the control and preeclampsia groups. Age seems to be a risk factor for preeclampsia, since women older than 40 years and younger than 20 years are at higher risk of preeclampsia. Being pregnant for the first time, among young women, may also be a factor that increases the risk of preeclampsia among these patients.^21^ In the control group, six patients were overweight (BMI from 25 kg/m^2^ to 29.9 kg/m^2^) and four patients were obese (BMI ≥ 30.0 kg/m^2^), while in the preeclampsia group, five patients were overweight and six were obese. Obesity is a major risk factor for preeclampsia, given that an increase in BMI of 7 kg/m^2^ doubles the risk of disease.^22^

Soonthornpun et al. demonstrated that even with no significant difference in BMI, women with preeclampsia had higher serum triglycerides and lower HDL levels than seen in the control group.^23^ Villa et al. investigated the fatty acid profile in women with preeclampsia and controls, and found that even without a difference in BMI, the preeclampsia group showed higher levels of oleic acid, linoleic acid and arachidonic acid, and increased insulin resistance.^19^

Preterm delivery (defined as gestational age < 37 weeks) is a common complication in preeclampsia patients (15-67%).^3^ As expected, in this study, the mean gestational age at delivery was lower in the preeclampsia group. However, this did not seem to influence our results.

Preeclampsia is one of the most important diseases during pregnancy, but its molecular mechanisms are not fully understood yet.^24^ Since the role of the placenta in the pathogenesis of preeclampsia is undisputed, and Akt/PBK action in insulin resistance is evident,^25-28^ we analyzed the expression of Akt/PKB at the baseline and with insulin stimulation, in placental tissue from preeclampsia and normal pregnancies. After insulin binding, the receptor is autophosphorylated, and it phosphorylates cytoplasmic substrates such as insulin receptor substrate 1 and PI3 kinase (PI3K). After phosphorylation, PI3K triggers phosphorylation of Akt/PKB at serine (ser473) and threonine (thr308) sites.^29^ Phosphorylated Akt/PKB translocates to the cytoplasm or to the nucleus, where it activates substrates with different actions on metabolism, growth and cell survival.^13^ Scioscia et al. showed that there was a decrease in tyrosine phosphorylation of IRS1, in insulin-stimulated preparations of human placenta from preeclampsia patients, compared with normal placenta, thus demonstrating that there is a lower level of insulin signaling in women with preeclampsia.^9^

In our study, sample stimulation was confirmed when the expression of phospho-Akt/PKB (p-Ser 473) was compared between insulin-stimulated and non-stimulated placenta cells. This signaling transduction pathway is an important milestone in the molecular mechanisms for insulin resistance syndrome in patients with preeclampsia.^9^ Data from Scoscia suggested that there was significantly higher serine phosphorylation of IRS1 and IRS2 in insulin-treated samples from preeclampsia patients. On the other hand, the insulin signal transduction signal was impaired in the controls,^9^ as also described in other insulin resistant states (diabetes type 2). Kunjara et al. demonstrated that accumulation of inositol phosphoglycan (P-IPG), a putative second insulin messenger, exerted several insulin-mimetic actions in placental tissue from preeclampsia patients and might be associated with insulin resistance.^30^

Orcy et al. previously studied the Akt/PKB expression pathway in the baseline state in the placenta, skeletal muscle and adipose tissue of preeclampsia patients. However, their findings did not show any significant difference in protein between the groups of patients.^6^ Similarly, our results also showed that there were no significant differences in baseline protein expression of Akt/PKB between the placentas of preeclampsia patients and controls. Likewise, there was no difference in stimulated Akt/PKB phosphorylation.

## CONCLUSIONS

*In vitro* stimulation of the human placenta using insulin has been well established. The expression of Akt/PKB in preeclampsia patients and controls, both at the baseline and in insulin-stimulated placenta samples, was similar. This suggests that there is similar activity of this pathway in these groups of women. Nevertheless, it cannot be ruled out that the Akt/PKB signaling pathway may have a role in the pathophysiology of preeclampsia, since the phosphorylation of Akt/PKB substrates still needs to be investigated.

## References

[1] Grill S Rusterholz C Zanetti-Dällenbach R. Potential markers of preeclampsia --a review. Reprod Biol Endocrinol. 2009;7:70. 19602262 10.1186/1477-7827-7-70 PMC2717076

[2] Milne F Redman C Walker J. The pre-eclampsia community guideline (PRECOG): how to screen for and detect onset of pre-eclampsia in the community. BMJ. 2005;330(7491):576-580. 15760998 10.1136/bmj.330.7491.576 PMC554032

[3] Sibai B Dekker G Kupferminc M. Pre-eclampsia. Lancet. 2005;365(9461):785-799. 15733721 10.1016/S0140-6736(05)17987-2

[4] Yung HW Calabrese S Hynx D. Evidence of placental translation inhibition and endoplasmic reticulum stress in the etiology of human intrauterine growth restriction. Am J Pathol. 2008;173(2):451-462. 18583310 10.2353/ajpath.2008.071193 PMC2475782

[5] Scioscia M Gumaa K Rademacher TW. The link between insulin resistance and preeclampsia: new perspectives. J Reprod Immunol. 2009;82(2):100-105. 19628283 10.1016/j.jri.2009.04.009

[6] Orcy RB Schroeder S Martins-Costa SH. Signalization of Akt/PKB in the placenta, skeletal muscle and adipose tissue of preeclampsia patients. Gynecol Obstet Invest. 2008;66(4):231-236. 18645256 10.1159/000147169

[7] Roberts JM. Preeclampsia: is there value in assessing before clinically evident disease? Obstet Gynecol. 2001;98(4):596-599. 11576574 10.1016/s0029-7844(01)01594-0

[8] Kossenjans W Eis A Sahay R Brockman D Myatt L. Role of peroxynitrite in altered fetal-placental vascular reactivity in diabetes or preeclampsia. Am J Physiol Heart Circ Physiol. 2000;278(4):H1311-H1319. 10749729 10.1152/ajpheart.2000.278.4.H1311

[9] Scioscia M Gumaa K Kunjara S. Insulin resistance in human preeclamptic placenta is mediated by serine phosphorylation of insulin receptor substrate-1 and -2. J Clin Endocrinol Metab. 2006;91(2):709-717. 16332940 10.1210/jc.2005-1965

[10] Hubbard SR Till JH. Protein tyrosine kinase structure and function. Annu Rev Biochem. 2000;69:373-398. 10966463 10.1146/annurev.biochem.69.1.373

[11] Taniguchi CM Emanuelli B Kahn CR. Critical nodes in signalling pathways: insights into insulin action. Nat Rev Mol Cell Biol. 2006;7(2):85-96. 16493415 10.1038/nrm1837

[12] Scotlandi K Picci P. Targeting insulin-like growth factor 1 receptor in sarcomas. Curr Opin Oncol. 2008;20(4):419-427. 18525338 10.1097/CCO.0b013e328302edab

[13] Andjelković M Maira SM Cron P Parker PJ Hemmings BA. Domain swapping used to investigate the mechanism of protein kinase B regulation by 3-phosphoinositide-dependent protein kinase 1 and Ser473 kinase. Mol Cell Biol. 1999;19(7):5061-5072. 10373555 10.1128/mcb.19.7.5061 PMC84347

[14] Klein HH Freidenberg GR Kladde M Olefsky JM. Insulin activation of insulin receptor tyrosine kinase in intact rat adipocytes. An in vitro system to measure histone kinase activity of insulin receptors activated in vivo. J Biol Chem. 1986;261(10):4691-4697. 3007472

[15] Bradford HF Richards CD. Specific release of endogenous glutamate from piriform cortex stimulated in vitro. Brain Res. 1976;105(1):168-172. 1252952 10.1016/0006-8993(76)90933-1

[16] Beisiegel U Weber W. Neuere Erkenntnisse zur Pathogenese der Familiären Hypercholesterinämie [New knowledge of the pathogenesis of familial hypercholesterolemia]. Verh Dtsch Ges Inn Med. 1986;92:383-389. 2949446

[17] Gershoni JM Palade GE. Protein blotting: principles and applications. Anal Biochem. 1983;131(1):1-15. 6193725 10.1016/0003-2697(83)90128-8

[18] Carty DM Delles C Dominiczak AF. Novel biomarkers for predicting preeclampsia. Trends Cardiovasc Med. 2008;18(5):186-194. 18790389 10.1016/j.tcm.2008.07.002 PMC2577131

[19] Villa PM Laivuori H Kajantie E Kaaja R. Free fatty acid profiles in preeclampsia. Prostaglandins Leukot Essent Fatty Acids. 2009;81(1):17-21. 19497719 10.1016/j.plefa.2009.05.002

[20] Scioscia M Greco P Selvaggi LE Rademacher TW. Is there a link between insulin resistance and inflammatory activation in preeclampsia?. Med Hypotheses. 2009;73(5):813-817. 19443128 10.1016/j.mehy.2009.01.057

[21] Lie RT Rasmussen S Brunborg H. Fetal and maternal contributions to risk of pre-eclampsia: population based study. BMJ. 1998;316(7141):1343-1347. 9563982 10.1136/bmj.316.7141.1343 PMC28531

[22] O'Brien TE Ray JG Chan WS. Maternal body mass index and the risk of preeclampsia: a systematic overview. Epidemiology. 2003;14(3):368-374. 12859040 10.1097/00001648-200305000-00020

[23] Soonthornpun K Soonthornpun S Wannaro P Setasuban W Thamprasit A. Insulin resistance in women with a history of severe pre-eclampsia. J Obstet Gynaecol Res. 2009;35(1):55-59. 19215548 10.1111/j.1447-0756.2008.00865.x

[24] Kaaja R Laivuori H Laakso M Tikkanen MJ Ylikorkala O. Evidence of a state of increased insulin resistance in preeclampsia. Metabolism. 1999;48(7):892-896. 10421232 10.1016/s0026-0495(99)90225-1

[25] Haruta T Morris AJ Vollenweider P. Ligand-independent GLUT4 translocation induced by guanosine 5’-O-(3-thiotriphosphate) involves tyrosine phosphorylation. Endocrinology. 1998;139(1):358-364. 9421434 10.1210/endo.139.1.5698

[26] Carpenter CL Duckworth BC Auger KR. Purification and characterization of phosphoinositide 3-kinase from rat liver. J Biol Chem. 1990;265(32):19704-19711. 2174051

[27] Auger KR Serunian LA Soltoff SP Libby P Cantley LC. PDGF-dependent tyrosine phosphorylation stimulates production of novel polyphosphoinositides in intact cells. Cell. 1989;57(1):167-175. 2467744 10.1016/0092-8674(89)90182-7

[28] Lee WJ. Insulin-like growth factor-I-induced androgen receptor activation is mediated by the PI3K/Akt pathway in C2C12 skeletal muscle cells. Mol Cells. 2009;28(5):495-499. 19855934 10.1007/s10059-009-0142-8

[29] Engelman JA Luo J Cantley LC. The evolution of phosphatidylinositol 3-kinases as regulators of growth and metabolism. Nat Rev Genet. 2006;7(8):606-619. 16847462 10.1038/nrg1879

[30] Kunjara S Greenbaum AL Wang DY. Inositol phosphoglycans and signal transduction systems in pregnancy in preeclampsia and diabetes: evidence for a significant regulatory role in preeclampsia at placental and systemic levels. Mol Genet Metab. 2000;69(2):144-158. 10720442 10.1006/mgme.2000.2964

